# Effectiveness of interventions to alleviate emergency department crowding by older adults: a systematic review

**DOI:** 10.1186/s12873-019-0288-4

**Published:** 2019-11-20

**Authors:** Gijs Hesselink, Özcan Sir, Yvonne Schoon

**Affiliations:** 1 Emergency Department, Radboud University Medical Center, Scientific Institute for Quality of Healthcare (IQ healthcare), Radboud Institute for Health Sciences, P.O. Box 9101, 114 IQ healthcare, Nijmegen, HB 6500 The Netherlands; 2 Radboud University Medical Center, Radboud Institute for Health Sciences, IQ health care, P.O. Box 9101, 114 IQ healthcare, Nijmegen, HB 6500 the Netherlands; 3 Department of Geriatrics, Radboud university medical center, Nijmegen, The Netherlands

**Keywords:** Systematic review, Older adults, Emergency department crowding

## Abstract

**Background:**

The growing demand for elderly care often exceeds the ability of emergency department (ED) services to provide quality of care within reasonable time. The purpose of this systematic review is to assess the effectiveness of interventions on reducing ED crowding by older patients, and to identify core characteristics shared by successful interventions.

**Methods:**

Six major biomedical databases were searched for (quasi)experimental studies published between January 1990 and March 2017 and assessing the effect of interventions for older patients on ED crowding related outcomes. Two independent reviewers screened and selected studies, assessed risk of bias and extracted data into a standardized form. Data were synthesized around the study setting, design, quality, intervention content, type of outcome and observed effects.

**Results:**

Of the 16 included studies, eight (50%) were randomized controlled trials (RCTs), two (13%) were non-RCTs and six (34%) were controlled before-after (CBA) studies. Thirteen studies (81%) evaluated effects on ED revisits and four studies (25%) evaluated effects on ED throughput time. Thirteen studies (81%) described multicomponent interventions. The rapid assessment and streaming of care for older adults based on time-efficiency goals by dedicated staff in a specific ED unit lead to a statistically significant decrease of ED length of stay (LOS). An ED-based consultant geriatrician showed significant time reduction between patient admission and geriatric review compared to an in-reaching geriatrician.

**Conclusion:**

Inter-study heterogeneity and poor methodological quality hinder drawing firm conclusions on the intervention’s effectiveness in reducing ED crowding by older adults. More evidence-based research is needed using uniform and valid effect measures.

**Trial registration:**

The protocol is registered with the PROSPERO International register of systematic reviews: ID = CRD42017075575).

## Background

Crowding is a constant and persistent phenomenon for the majority of EDs around the world [1, 2]. ED crowding can be defined as a situation in which the demand for emergency services exceeds the ability of physicians and nurses to provide quality care within a reasonable time [3, 4]. This phenomenon often occurs when EDs need to care for a greater number of patients than they are ideally designed for [3]. Consequently, ED crowding is associated with increased adverse clinical outcomes [5–10], care delays [6, 11–13], patient dissatisfaction with emergency care, [13] higher left without being seen rates [6, 14], and an increased chance of avoidable and costly hospital readmissions [9, 15].

Findings of a systematic review of 102 studies by Morley et al. show that older people at the ED have a significant negative impact on ED crowding [16]. Older people are often attending the ED with atypical and psychosocial problems that can complicate the provision of appropriate and timely ED care [17]. Despite the clear need to reduce ED crowding by older adults, a comprehensive evaluation of the effectiveness of interventions targeting this problem is lacking. Several reviews have studied the nature and effectiveness of interventions for older patients in need of emergency care [18–20]. However, McCusker and Verdon evaluated a specific type of intervention (i.e., comprehensive geriatric assessment; CGA) on a single effect measure (i.e., ED visits) [18]. Fan et al. reviewed the effectiveness of interventions targeting the older adult population in reducing ED utilization [19]. Effects of interventions on other relevant ED crowding indicators (e.g., waiting time, ED boarding time) therefore remain unknown. The review by Aminzadeh and Dalziel was conducted more than a decade ago, included studies with weak designs and did not specifically evaluate the intervention’s impact on ED crowding [20].

Better insight into interventions that reduce crowding by older adults in the ED is needed to assist managers and healthcare providers in emergency medicine worldwide with deliberately selecting and implementing strategies based on available evidence. Therefore, our aim is to systematically review the effectiveness of interventions targeting the older adults in reducing ED crowding, and identify core characteristics shared by successful intervention models.

## Methods

We planned and reported this systematic review in accordance with the guideline for performing and reporting systematic reviews and meta-analyses (PRISMA) [21]. The protocol of this review is accessible on the PROSPERO website (registration number: CRD42017075575).

### Data sources and searches

We searched for studies published between January 1990 and March 2017 in the following databases: PubMed (including MEDLINE), Cumulative Index to Nursing and Allied Health Literature (CINAHL), the Cochrane Library, EMBASE and PsychInfo. Our search strategies comprised a combination of key search terms related to the ‘emergency department’, ‘elderly patients’, ‘(quasi) experimental studies’, and ED crowding measures (see Additional file 1). References of the selected publications were manually checked to identify additional relevant studies that were missed in the database search (snowballing). We also searched for additional relevant studies in the online archives/bibliographies of three high-impact journals in the field of emergency care (i.e., *Annals of Emergency Medicine, Injury, Academic Emergency Medicine)*.

### Study selection

Studies were included if they were: 1) published with an abstract in English language; 2) used an experimental or quasi-experimental design (i.e., RCT, non-RCT, CBA, time-series); 3) evaluated an intervention targeting older adults (≥ 60 years of age); and 4) reported outcome effects on ambulance diversion, waiting time or count, patient leaves before treatment, ED occupancy level, time to consultation or ED room/bed placement, ED LOS, ED boarding time or count, ED return visits or ED staff stress level. We defined these ‘direct’ outcome measures based on the input-throughput-output model for ED crowding by Asplin et al. [22], the outcomes of a systematic review on ED crowding measures [23], expert opinions of circumstances that define ED crowding [24], and the consensus definition of crowding by Boyle et al. [2]. Table 1 shows the definitions for each type of measure. ED return visits and the ED staff stress level are considered to be important ‘indirect’ indicators for ED crowding [22, 23]. ED revisits may indirectly contribute to an increase of the ED input that is higher than the ED staff can handle. We also included intervention studies with a different aim (e.g., improving patient health outcomes following an ED visit) as long as we could identify that crowding reduction was a secondary study aim and if effectiveness was assessed on one or more of the above mentioned relevant outcome measures.

**Table 1:** Direct and indirect measures of crowding in the ED

Direct measures			Indirect measures
*Input*	*Throughput*	*Output*	
Ambulance diversion^a^	Occupancy level^e^	Boarding time^i^	ED staff stress level^k^
Waiting time^b^	Time to consultation^f^	Boarding count^j^	Return visits^l^
Waiting count^c^	Time to ED room/bed placement^g^		
Leaves without being seen^d^	Length of stay^h^		

^a^ EDs diverting ambulances due to capacity problems

^b^ Time between arrival on the ED and initial triage

^c^ Number, percentage or mean of patients in the ED waiting room

^d^ Patient leaves of the ED before start or completion of the treatment

^e^ Volume of patients in the ED compared to the number of officially designated ED spaces, waiting or treatment rooms

^f^ Time between registration at the ED and the first visit of an emergency physician or relevant subspecialist (e.g., geriatrician)

^g^ Time between ED registration or initial triage and placement in an ED treatment room or bed

^h^ Time between arrival on the ED and discharge, admittance on a ward or death

^i^ Time patients are hold in the ED after the admission decision

^j^ Number, percentage or mean of patients in the ED after the admission decision

^k^ Work-related feelings of stress (e.g., fatigue, burnout, being rushed) by emergency care physicians and nurses

^l^ ED visits after index visit which may be the consequence of patient leaves without being seen or poor discharge due to time restraints and limited possibilities to arrange appropriate follow-up care

Two reviewers (G.H. and J.v.H.) independently assessed inclusion eligibility of the retrieved studies using the search strategy. The initial selection for inclusion was based on the title and abstract of the study. When the title and abstract provided insufficient information to determine the relevance, a full-text copy of the article was retrieved and reviewed. For the final selection, a full-text copy of the study was examined to determine whether it fulfilled the inclusion criteria. Disagreement about inclusion was solved by discussion. When no consensus was achieved, a third reviewer (Y.S.) made the final decision.

### Data extraction

Two reviewers (G.H. and J.v.H.) independently extracted data from included studies. A standardized form was used to ensure consistency of data extraction. The following data were extracted from individual studies: country, publication year, study design, study setting, population characteristics, sample size, intervention details and the observed outcome effects on measures of interest.

### Assessment of risk of bias in included studies

Two reviewers (G.H. and J.v.H.) independently rated methodological quality. Studies were assessed using the suggested risk of bias criteria for Cochrane Effective Practice and Organisation of Care (EPOC) reviews [25]. We assessed studies for generation of allocation sequence, concealment of allocation, similar baseline outcome measurements, similar baseline characteristics, incomplete outcome data, blinding of participants, blinding of outcome assessors, protection against contamination, selective outcome reporting and other risks of bias. The decision on whether the criteria were fulfilled was resolved by discussion, or consulting a third researcher (Y.S.). As suggested by Davey et al. [26], we scored each study for risk of bias as ‘Low’ if all criteria were scored as ‘Low risk’, ‘Moderate’ if one or two criteria were scored as ‘Unclear’ or ‘High risk’, and ‘High’ if more than two criteria were scored as ‘Unclear’ or ‘High risk’. Inter-rater agreement for the individual domains of the risk of bias was calculated by between-group Kappa agreement, using the assessments from each reviewer before resolution of disagreements.

### Data synthesis and analysis

Study outcomes were organized in tabular form and a qualitative assessment was made based on the methodological quality, intervention characteristics, outcomes, statistical significance, and direction of effects observed. Core elements of interventions were identified in a similar approach to Sinha et al. [27], and Fan et al. [20]. One primary reviewer (G.H.) reviewed each study and listed all characteristic elements included in the studied interventions. Individual studies were further examined to determine whether they were using the same terms to describe different elements or different terms to describe the same elements. This process ultimately enabled a set of core elements. Subsequently, each individual intervention was again reviewed, determined as adhering or not to a particular category. Previous steps were checked by a second reviewer (Y.S.), an experienced geriatrician, and discrepancies were resolved by consensus. If possible, summaries of intervention effects for each study were provided by calculating risk ratios (RR) for dichotomous outcomes and standardised mean differences (SMD) for continuous outcomes. We performed a meta-analysis using the Review Manager 5 data analysis programme when two or more studies were RCTs and the outcome measures and type of intervention could be compared. If no significant heterogeneity was present (I^2^ < 70%) [28], a random-effects meta analysis was performed of binary (e.g. ED revisits) and continuous (e.g. ED LOS) outcomes. Statistical significance was set at *p* < 0.05.

## Results

### Search results

**Fig. 1:**
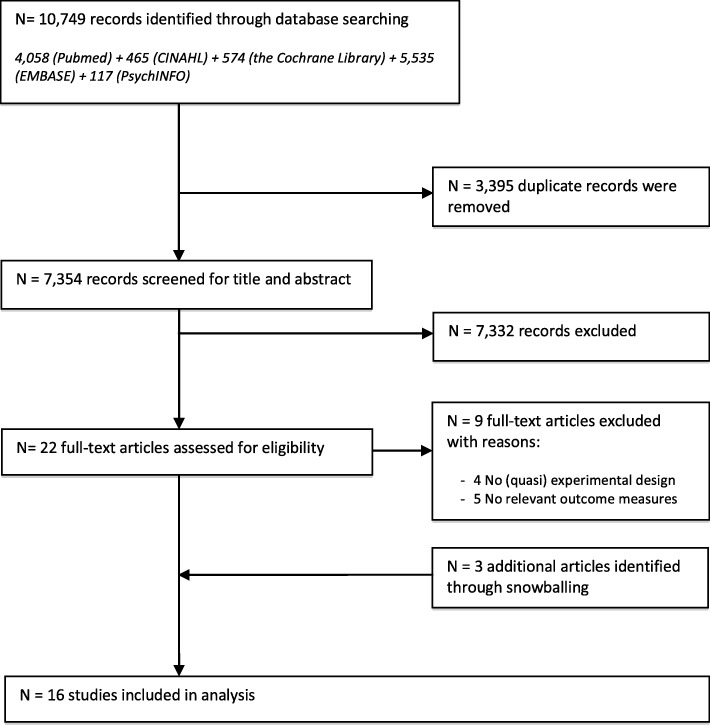
Flow chart of the study selection and review process

Our initial search identified 10,749 records. After exclusion of duplicates 7354 records were screened by title and abstract. Twenty-two full-text studies were retrieved and reviewed of which nine were excluded. Three studies were identified through snowballing. The final set consisted of 16 studies that underwent full-data extraction (Fig. 1).

### Study characteristics

The characteristics of the included studies are summarized in an additional file (see Additional file 2). Eight RCT’s [29–36], six CBA studies [37–42], and two NRCT’s [43, 44], fulfilled our inclusion criteria. Of the 16 included studies, five were conducted in the United States [32, 36, 39, 41, 43], four in Canada [29, 31, 34, 37], three in the United Kingdom [30, 40, 42], three in Australia [33, 35, 44], and one in Singapore [38]. The studies were published between 1996 and 2016 (median publication year = 2010; IQR = 2003 to 2013).

Despite the shared focus on the older adult patient population, included studies varied in population by age, health condition, and place and time of exposure to the intervention. Of the 16 studies, 10 included patients aged ≥65 years [30–32, 35, 36, 38, 40, 41, 43, 44], three included patients aged ≥75 years [33, 37, 42], two included patients aged ≥70 years [29, 34], and one included patients aged ≥60 years [39]. Seven studies further specified their inclusion criteria to: patients admitted to the ED with a medical diagnosis [35], a chronic disease [44], a traumatic injury [39], fall injuries [30, 34], increased risk of ED readmission [29, 35], and requiring outpatient follow-up – like assistance with activities of daily living (ADL) at home – after ED discharge [29, 36]. The sample size ranged from 43 to 3748 participants for the intervention groups and from 43 to 3850 participants for the control groups.

Of the 16 studies, four (25%) focused on measuring ED throughput time: i.e., ED LOS [39, 43, 44], and time until patients are reviewed by a geriatrician [42]. Effect on ED throughput time was the primary outcome in three studies [39, 42, 44]. Thirteen studies compared the ED revisit rates for intervention groups with the controls [29–38, 40–42]. Follow-up measurement periods varied within and between studies from 7 days to 18 months after the patient’s initial ED visit.

### Risk of bias in included studies

**Fig. 2:**
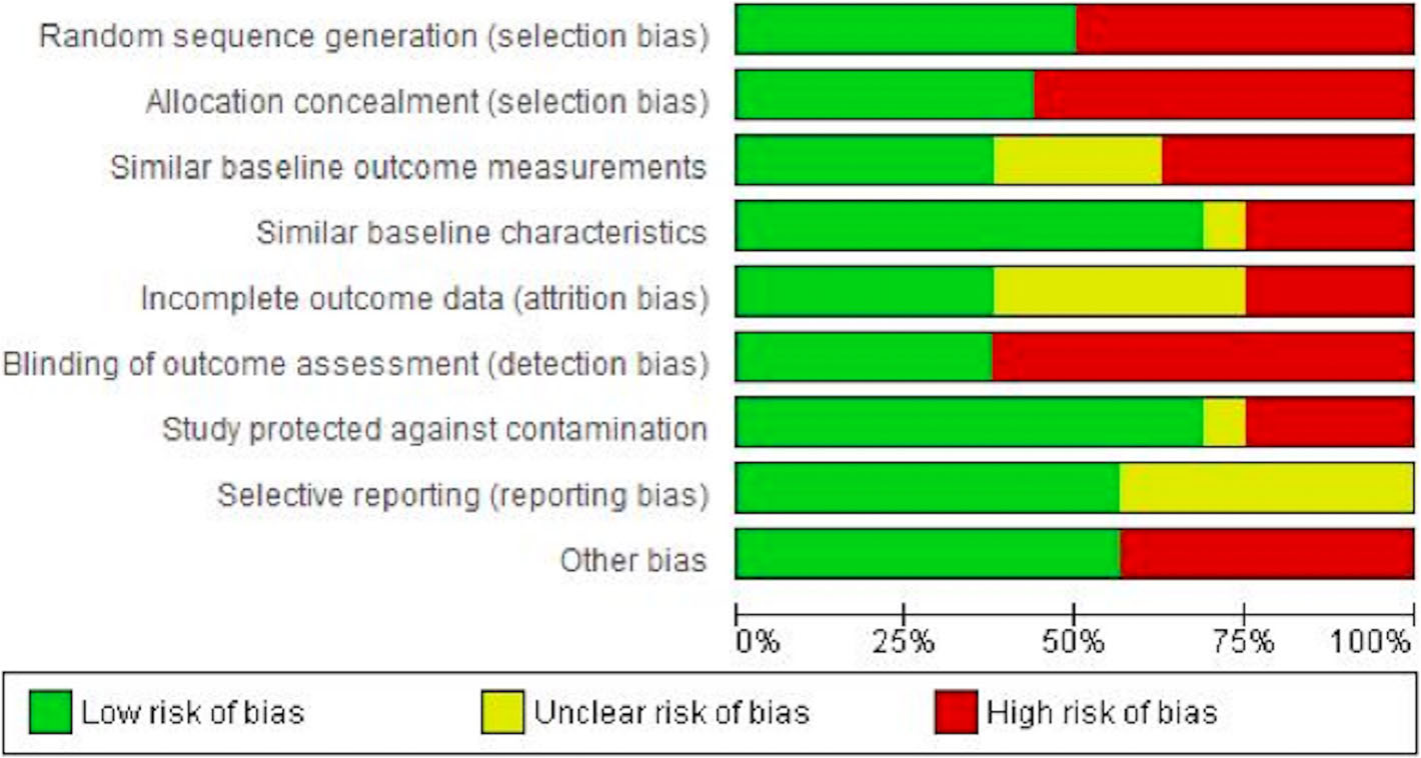
Reviewers' judgments about each risk of bias item presented as percentages across all included studies; legend: Green (low risk of bias); Yellow (unclear risk of bias); Red (high risk of bias)

Overall judgement scores on each risk of bias item are presented in Fig. 2. Of the 16 studies, twelve studies (75%) had a high risk of bias, most commonly due to inadequate randomization, allocation concealment, and blinding [30, 31, 33, 36–44]. Two studies (13%), both RCTs, had a low risk of bias [29, 34]. Two other RCTs (13%) had a moderate risk of bias [32, 35]. The reviewers could not ascertain whether one study was protected against contamination [35], and whether two studies were free from selective outcome reporting [32, 35]. Inter-rater agreement for the individual domains of risk of bias varied – before resolution of disagreements – between slight agreement for ‘Study protected against contamination’ (kappa of 0.30) and very good agreement for ‘Random sequence generation’ (kappa of 0.88).

#### Intervention characteristics

Based on our qualitative assessment, seven core elements central to the studied interventions were identified and adherence of each intervention to these elements are summarized (see additional file 3), with all studies being organized according to the primary setting of their investigated interventions, namely: the hospital setting and the community setting. Eleven studies examined interventions that were implemented in an hospital setting [31, 32, 35–44]. In the other five studies patients were primarily exposed to the intervention in the community, mostly at home [29, 30, 33, 34, 36]. Ten interventions consisted of a geriatrician or geriatric pharmacist embedded within the ED, or ED staff with geriatric expertise to facilitate more efficient and effective care for older adults [29, 32, 33, 37, 38, 40–44]. Ten interventions incorporated case management by multidisciplinary teams that assessed patient-specific needs, provided care strategies and linked patients to necessary services [29, 31–33, 35, 38–42]. Ten interventions initiated care and disposition planning by regular ED staff or nurse liaisons to ensure continuity of care after a patient’s discharge from the ED [29, 31–33, 35, 37, 38, 41, 43, 44]. Nine interventions provided post-ED discharge follow-up, mostly through telephone calls by ED nurses [31, 32, 35–37, 43], and by home visits to manage care needs and link patients to required services [29, 30, 33]. Eight interventions included ED-based geriatric assessment to improve a timely recognition and treatment of geriatric problems. Five of the eight interventions consisted of CGA [32, 40–43]. Four interventions were specific units or zones with dedicated space and beds to address the specific emergency needs of older adults [39–42]. Taylor et al. [42], and Conroy et al. [40], created an ‘acute frailty zone’ within the ED – with early access to geriatrician-led multidisciplinary input and CGA – that replaced the pre-existing geriatrician in-reach service. Lee et al. [34], described a portable Personal Emergency Response System (PERS) for patients who recently visited the ED after a fall incident. This intervention did not adhere to any of the intervention core elements.

### Intervention effects on ED throughput time

One CBA study, with high risk of bias, reported a significant reduction of ED LOS for patients who were treated at a geriatric trauma unit compared to the control group [39]. Patients presented at the unit were assessed by an ED physician on established criteria for geriatric trauma service activation. Upon activation patients were seen immediately by the trauma service and a hospitalist, and quickly by relevant ancillary services. The service was organized around efficiency time-to-care goals for the medical staff. Trauma surgeons acted as coordinators for the older trauma patient to facilitate definitive management of the injury by subspecialists [39]. On the contrary, two other studies reported a significant longer ED LOS for patients who were exposed to the intervention [43, 44]. Miller and colleagues performed a RCT to measure the effects of a combined ED-based CGA, liaison services and telephone follow-up by a geriatric nurse [43]. Patients in the intervention group stayed on average 1 h longer at the ED compared to patients receiving usual care. In a NRCT, Mortimer and colleagues found that older adults who were seen at the ED by a geriatric pharmacist stayed on average 2.6 h longer than older adults receiving usual care management at the ED [44]. Both studies suffered from a high risk of bias (e.g., no randomization, allocation concealment, blinding of outcome assessors, possible contamination between intervention and control groups). Furthermore, one CBA study reported that older adults in an embedded frailty emergency zone with a dedicated consultant geriatrician were seen and reviewed significantly earlier (twice as fast) by a geriatrician than controls receiving emergency care by an in-reaching geriatrician service [42].

### Intervention effects on ED revisits

Nine studies reported a decrease in ED revisit rates for intervention groups compared to the controls [30, 32, 33, 35–37, 40–42]. One RCT, with moderate risk of bias, reported a significant decrease of revisits within 6 months post index ED visit for discharged older adults following a 24-week home-based exercise and telephone follow-up program compared to controls receiving usual emergency care and discharge planning [35]. On the contrary, one RCT with low risk of bias reported a significant increase in the average number of revisits within 10 months post index ED visit for older adults receiving community-based nurse case management compared to the controls receiving usual care [29].

### Meta analysis

The high risk of bias in most of the included studies and heterogeneity of the treatment effect (I^2^ > 70%), differences in follow-up measurement periods, and the multi-component characteristics of most studied interventions hindered appropriate and reliable meta-analytic pooling for effect estimates.

## Discussion

To our knowledge, this is the first comprehensive systematic review of literature evaluating the effectiveness of interventions on reducing ED crowding by older adults. We identified two types of interventions that showed to be effective in alleviating ED crowding. First, the combination of initial triage of older adults by the ED physician and multidisciplinary care – according to time-efficiency goals – within a specific hospital-based geriatric emergency unit contributes to a reduced LOS of older adults in the ED. This finding corresponds with literature on improving ED patient flow. The use of doctor-led triage [45], rapid assessment [46], and streaming (i.e., allocating similar patient types to a particular work stream were they are assessed by dedicated staff in a specific geographical area within the ED) [47], have all been shown to improve patient flow and thus alleviate ED crowding. Second, older adults treated in an emergency care setting with an embedded geriatrician receive more timely geriatric assessment compared to an in-reaching geriatrician service. This finding is in line with previous studies addressing the value of putting geriatricians at the “front door” of the hospital; it allows early specialist review, reduces the undertaking of multiple similar patient assessments by medical staff and improves the timeliness and appropriateness of ED disposition decisions [48]. Literature shows that many ED physicians and nurses are not well-trained in geriatric emergency medicine and feel less comfortable when dealing with older adults [49, 50]. Consequently, the management of older adults in the ED often requires more time and resources compared to younger adults [51]. The presence of a geriatrician could help ED staff in becoming more capable and confident in dealing with older adults in a timely manner.

Despite these positive findings, robust evidence for effective interventions in alleviating ED crowding by the older patient population remains limited. Significant effects are based on single studies, limiting the ability to generalize findings across ED settings. Moreover, individual studies with positive effects on reducing crowding are not supported by other studies evaluating a similar type of intervention on the same outcome. Many interventions showed reduced ED revisits for older adults, but lacked statistical significance. Some interventions also demonstrated opposite effects, such as a prolonged ED LOS and increase of ED revisits. These effects may be explained by the time needed to carry out the intervention (e.g., an ED-based geriatric nurse of pharmacist responsible for cross-checking medications and organizing appropriate referrals).

Although ED crowding by older adults is considered to be a global problem and threat to patient safety [16], the amount and quality of experimental research dedicated to this urgent problem is surprisingly poor. We found only one CBA study that explicitly addressed the problem of crowding as the leading motive for intervention development and testing [37]. Moreover, only four studies evaluated intervention effects on ED throughput efficiency (e.g., ED LOS, time to geriatric review). Studies evaluating interventions on two other important components for explaining ED crowding – ED input and output efficiency were not found. These findings call for a more valid and comprehensive evaluation of interventions targeting ED crowding reduction by older adults visiting the ED. Our operationalisation of ED crowding measures (Table 1) and the overviews of measures provided by others [2, 22–24], may guide researchers in selecting uniform and valid outcomes. In addition to the measurement of effects, more insight is needed into the feasibility of interventions and the factors that hinder and promote successful implementation to better inform policy-makers on selecting and implementing interventions based on the local needs and possibilities. For example, the introduction of a geriatric emergency unit, efficiency goals and embedding a geriatrician at the ED may involve significant costs and changes in work routines. The commitment from many different medical specialties and strong leadership may then be important factors determining the intervention’s success [39].

### Study limitations

Our review had several limitations. First, we used a wide set of internationally accepted measures of ED crowding to objectively assess publications on their relevance. However, to date, there are no uniform criteria to define and measure ED crowding. As a consequence, potentially relevant studies using other measures for ED crowding might have been overlooked.

Second, marked heterogeneity among studies, particularly in interventions and outcome measurement periods, precluded meta-analysis and made it difficult to draw firm conclusions. Second, comparison of effects between studies were hindered by varying population groups. Among the 16 included studies, four different thresholds of old age were used to mark the older patient. Some studies focused on subgroups of older adults (i.e., with a chronic disease, with a fall history and with a traumatic injury). Third, comparison of effects between studies were hindered because the majority of studies implemented and evaluated an intervention within a single institution. Study findings may be difficult to compare because of differences in the organizational structure (e.g., access or systems for referral and follow-up of patients), work routines, bed-capacity and the geographical location of studied ED sites. Also, it is possible that interventions’ measured effects in reducing ED revisits were underestimated in single-institution studies if older adults shifted their visits to other ED sites. Fourth, overall risk of bias was high in most of the included studies. In several studies the reliability of outcome effects may be negatively affected by patient’s self-reported ED revisits. Therefore, individual study findings need to be interpreted with caution. Fifth, we performed an additional search in three specific journals to broaden our search for relevant publications on the abstract phenomenon of crowding. However, using this journal-specific search strategy may have introduced bias in the selection of studies. Finally, we did not include studies published outside of the peer-reviewed scientific literature. Publication bias may have affected our results.

## Conclusion

Given the global aging population and its impact on the growing problem of ED crowding, there is an urgent need to focus future research on intervention studies aimed at improving the organization and efficiency of care for older adults in the ED. The rapid assessment of older patients and streaming of care based on time-efficiency goals, and an ED-based consultant geriatrician seem to be promising strategies for alleviating ED crowding by this specific patient group. Ultimately, this must lead to better quality of care and better health outcomes for older patients in the ED. However, the poor methodological quality, the differences in intervention types and used outcome effects, and the validity of used outcome measures hinder the demonstration of robust evidence to support these interventions. Our hope is that this systematic review will act as a stimulus for conducting more high-quality experimental research on reducing ED crowding by older adults, using uniform and valid effect measures to ensure generalisability in the evaluation of the true effectiveness of interventions.

## Supplementary information

**Additional file 1.** Search strategy. Detailed database-specific search strings.

**Additional file 2.** Characteristics of included studies.

**Additional file 3.** Studies organized according to type of intervention and outcome.

## Data Availability

The datasets used and analysed during the current study are available from the corresponding author on reasonable request.

## Abbreviations

ADL: Activities of Daily Living
CBA: Controlled Before-After
CGA: Comprehensive Geriatric Assessment
CINAHL: Cumulative Index to Nursing and Allied Health Literature
ED: Emergency Department
EPOC: Effective Practice and Organisation of Care
LOS: Length Of Stay
PERS: Personal Emergency Response System
PRISMA: Performing and Reporting Systematic Reviews and Meta-Analyses
RCT: Randomized Controlled Trial
RR: Risk Ratios
SMD: Standardised Mean Differences
