# Supplementary material for: Effectiveness of interventions to alleviate emergency department crowding by older adults: a systematic review
Source: BMC Emerg Med. 2019 Nov 20;19:69. doi: 10.1186/s12873-019-0288-4 (PMC6864956; doi:10.1186/s12873-019-0288-4)
Supplement: Supplementary file 1 — Additional file 1. Search strategy. Detailed database-specific search strings. [file 12873_2019_288_MOESM1_ESM.pdf]

Pubmed (April 13, 2017)

Search strategy

((((((((emergency service, hospital [MeSH]) OR emergency medical services [MeSH]) OR emergency medicine [MeSH]) OR emergency department [tiab]) OR ER [tiab]) OR emergency room [tiab])) AND ((((((((((((randomized controlled trial [PT]) OR controlled clinical trial [PT]) OR evaluation studies [PT]) OR comparative study [PT]) OR experiment* [tiab]) OR control* [tiab]) OR compar* [tiab]) OR control group [tiab]) OR time series [tiab]) OR before after [tiab]) OR (before and after [tiab])) OR random* [tiab]) OR effect* [tiab])) AND (((((((aged [MeSH]) OR frail elderly [MeSH]) OR health services for the aged [MeSH]) OR elder* [tiab]) OR aged [tiab]) OR geriatric* [tiab]) OR older* [tiab]) OR senior* [tiab])) AND (((((((((((((boarding [tiab]) OR occupancy [tiab]) OR return visit* [tiab]) OR offload* [tiab]) OR patient leave* [tiab]) OR patient satisfaction [tiab]) OR length of stay [tiab]) OR ED bed* [tiab]) OR exit block [tiab]) OR crowding [MeSH]) OR crowding* [tiab]) OR overcrowding [tiab])) OR ((time [tiab]) AND (((((((((door [tiab]) OR registration [tiab]) OR floor [tiab]) OR physician [tiab]) OR doctor [tiab]) OR nurse [tiab]) OR admission [tiab]) OR discharge [tiab]) OR waiting [tiab]) OR triage [tiab])))

Hits: 4058

CINAHL (April 13, 2017)

Search strategy

S50 S7 AND S15 AND S23 AND S49

S49 S35 OR S48

S48 S46 AND S47

S47 S36 OR S37 OR S38 OR S39 OR S40 OR S41 OR S42 OR S43 OR S44 OR S45

S46 TX time

S45 TX waiting

S44 TX discharge

S43 TX admission

S42 TX nurse

S41 TX doctor

S40 TX physician

S39 TX floor

S38 TX registration

S37 TX door

S36 TX triage

S35 S24 OR S25 OR S26 OR S27 OR S28 OR S29 OR S30 OR S31 OR S32 OR S33 OR S34

S34 TX overcrowding

S33 TX crowding

S32 (MH "Crowding")

S31 TX exit block

S30 TX length of hospital stay

S29 TX patient satisfaction

S28 TX patient leave*

S27 TX offload*

S26 TX return visit*

S25 TX occupancy

S24 TX boarding

S23 S16 OR S17 OR S18 OR S19 OR S20 OR S21 OR S22

S22 AB senior*

S21 AB older*

S20 AB geriatric*

S19 AB aged

S18 AB elder*

S17 (MH "Health Services for the Aged")

S16 (MH "Aged+") OR (MH "Frail Elderly")

S15 S9 OR S10 OR S11 OR S12 OR S13 OR S14

S14 (MH "Evaluation Research+")

S13 (MH "Quasi-Experimental Studies+")

S12 (MH "Control Group")

S11 (MH "Control (Research)+")

S10 (MH "Comparative Studies")

S9 (MH "Clinical Trials+")

S8 ((TI Randomi?ed control$ trial$ or AB Randomi?ed control$ trial$)) OR (random assignment) OR (((TI control* or AB control*) and (TI random* or AB random*) and (TI trial* or AB trial*))) OR ((TI random* or AB random*) and (TI trial* or AB trial*) and (TI clinical* or AB clinical*))) OR PT clinical trial)

S7 S1 OR S2 OR S3 OR S4 OR S5 OR S6

S6 AB ER

S5 AB emergency room

S4 AB emergency department

S3 (MH "Emergency Medicine")

S2 (MH "Emergency Medical Services+")

S1 (MH "Emergency Service+")

Hits: 465

Cochrane library (April 13, 2017)

Search strategy

#1 emergency department:ti,ab,kw

#2 emergency room:ti,ab,kw

#3 emergency ward:ti,ab,kw

#4 emergency service:ti,ab,kw

#5 #1 or #2 or #3 or #4

#6 "randomised controlled trial":ti,ab,kw

#7 "randomised controlled trial":ti,ab,kw

#8 experimental:ti,ab,kw

#9 "quasi experimental study":ti,ab,kw

#10 "control":ti,ab,kw

#11 "control group":ti,ab,kw

#12 before after:ti,ab,kw

#13 ''before and after'':ti,ab,kw

#14 "effect":ti,ab,kw

#15 "random":ti,ab,kw

#16 randomised:ti,ab,kw

#17 effectiveness:ti,ab,kw

#18 #6 or #7 or #8 or #9 #10 or #11 or #12 or #13 #14 or #15 or #16 or #17

#19 aged:ti,ab,kw

#20 elderly:ti,ab,kw

#21 older:ti,ab,kw

#22 frail elderly:ti,ab,kw

#23 MeSH descriptor: [Aged] explode all trees

#24 MeSH descriptor: [Health Services for the Aged] explode all trees

#25 #19 or #20 or #21 or #22 or #23 or #24

#26 return visit*:ti,ab,kw

#27 patient leave*:ti,ab,kw

#28 patient satisfaction:ti,ab,kw

#29 length of stay:ti,ab,kw

#30 ED bed*:ti,ab,kw

#31 MeSH descriptor: [Crowding] explode all trees 27

#32 crowding:ti,ab,kw

#33 overcrowding:ti,ab,kw

#34 #26 or #27 or #28 or #29 or #30 or #31 or #32 or #33

#35 triage:ti,ab,kw

#36 waiting:ti,ab,kw

#37 discharge:ti,ab,kw

#38 admission:ti,ab,kw

#39 nurse:ti,ab,kw

#40 doctor:ti,ab,kw

#41 physician:ti,ab,kw

#42 floor:ti,ab,kw

#43 registration:ti,ab,kw

#44 #35 or #36 or #37 or #38 or #39 or #40 or #41 or #42 or #43

#45 time:ti,ab,kw

#46 #44 and #45

#47 #34 or #46

#48 #5 and #18 and #25 and #47

Hits: 574

EMBASE (April 13, 2017)

Search strategy

#1 exp emergency health service/

#2 exp emergency medicine/

#3 emergency department.ab,ti.

#4 emergency room.ab,ti.

#5 ER.ab,ti.

#6 controlled study/

#7 exp "randomized controlled trial (topic)"/

#8 exp quasi experimental study/ or exp experimental design/

#9 exp comparative study/

#10 "control*".ab,ti.

#11 control group.ab,ti.

#12 "experiment*".ab,ti.

#13 random.ab,ti.

#14 randomi?ed.ab,ti.

#15 "compar*".ab,ti.

#16 "effect*".ab,ti.

#17 time series.ab,ti.

#18 before after.ab,ti.

#19 (before and after).ab,ti.

#20 aged/

#21 exp geriatrics/

#22 exp aging/ or exp attitude to aging/

#23 exp very elderly/ or exp elderly care/

#24 "elder*".ab,ti.

#25 aged.ab,ti.

#26 "geriatric*".ab,ti.

#27 "older*".ab,ti.

#28 "senior*".ab,ti.

#29 "frail elder*".ab,ti.

#30 exp "crowding (area)"/

#31 crowding.ab,ti.

#32 overcrowding.ab,ti.

#33 boarding.ab,ti.

#34 occupancy.ab,ti.

#35 "return visit*".ab,ti.

#36 "ED return visit*".ab,ti.

#37 occupancy.ab,ti.

#38 "offload*".ab,ti.

#39 "patient leave*".ab,ti.

#40 patient satisfaction.ab,ti.

#41 length of stay.ab,ti.

#42 "ED bed*".ab,ti.

#43 exit block.ab,ti.

#44 door.ab,ti.

#45 registration.ab,ti.

#46 floor.ab,ti.

#47 physician.ab,ti.

#48 doctor.ab,ti.

#49 nurse.ab,ti.

#50 admission.ab,ti.

#51 discharge.ab,ti.

#52 waiting.ab,ti.

#53 triage.ab,ti.

#54 time.ab,ti.

#55 #44 or #45 or #46 or #47 or #48 or #49 or #50 or #51 or #52 or #53

#56 #54 and #55

#57 #1 or #2 or #3 or #4 or #5

#58 #6 or #7 or #8 or #9 or #10 or #11 or #12 or #13 or #14 or #15 or #16 or #17 or #18 or #19

#59 #20 or #21 or #22 or #23 or #24 or #25 or #26 or #27 or #28 or #29

#60 #30 or #31 or #32 or #33 or #34 or #35 or #36 or #37 or #38 or #39 or #40 or #41 or #42 or #43 or #56

#61 #57 and #58 and #59 and #60

Hits: 5,535

Psychinfo (April 13, 2017)

Search strategy

#1 exp Emergency Services/

#2 emergency department.ab,ti.

#3 emergency room.ab,ti.

#4 ER.ab,ti.

#5 #1 or #2 or #3 or #4

#6 exp EXPERIMENTAL DESIGN/

#7 exp Clinical Trials/

#8 random.ab,ti.

#9 randomi?ed.ab,ti.

#10 "compar*".ab,ti.

#11 "effect*".ab,ti.

#12 time series.ab,ti.

#13 before after.ab,ti.

#14 (before and after).ab,ti.

#15 "experiment*".ab,ti.

#16 "control*".ab,ti.

#17 control group.ab,ti.

#18 #6 or #7 or #8 or #9 or #10 or #11 or #12 or #13 or #14 or #15 or #16 or #17

#19 exp Geriatrics/

#20 exp Aging/

#21 exp Elder Care/

#22 exp "AGED (ATTITUDES TOWARD)"/

#23 "elder*".ab,ti.

#24 aged.ab,ti.

#25 geriatric*.ab,ti.

#26 "older*".ab,ti.

#27 "senior*".ab,ti.

#28 #19 or #20 or #21 or #22 or #23 or #24 or #25 or #26 or #27

#29 boarding.ab,ti.

#30 occupancy.ab,ti.

#31 "return visit*".ab,ti.

#32 "offload*".ab,ti.

#33 "patient leave*".ab,ti.

#34 patient satisfaction.ab,ti.

#35 length of stay.ab,ti.

#36 "ED bed*".ab,ti.

#37 exit block.ab,ti.

#38 exp CROWDING/

#39 crowding.ab,ti.

#40 overcrowding.ab,ti.

#41 #29 or #30 or #31 or #32 or #33 or #34 or #35 or #36 or #37 or #38 or #39 or #40

#42 door.ab,ti.

#43 registration.ab,ti.

#44 floor.ab,ti.

#45 physician.ab,ti.

#46 doctor.ab,ti.

#47 nurse.ab,ti.

#48 admission.ab,ti.

#49 discharge.ab,ti.

#50 waiting.ab,ti.

#51 triage.ab,ti.

#52 #42 or #43 or #44 or #45 or #46 or #47 or #48 or #49 or #50 or #51

#53 time.ab,ti.

#54 #52 and #53

#55 #41 or #54

#56 #5 and #18 and #28 and #55

Hits: 117
