# Supplementary material for: Effectiveness of interventions to alleviate emergency department crowding by older adults: a systematic review
Source: BMC Emerg Med. 2019 Nov 20;19:69. doi: 10.1186/s12873-019-0288-4 (PMC6864956; doi:10.1186/s12873-019-0288-4)
Supplement: Supplementary file 2 — Additional file 2. Characteristics of included studies. [file 12873_2019_288_MOESM2_ESM.doc]

| **Source (year); country** | **Design;**  **period** | **Primary outcome(s)** | **Sample characteristics** | **Results** | | | | |
| --- | --- | --- | --- | --- | --- | --- | --- | --- |
|  |  |  |  | ***Relevant outcome measure; follow-up period*** | ***I*** | ***C*** | ***RR (95% CI)*** | ***P<.05*** |
| Miller et al [43] (1996)  USA | NRCT;  11 mo | Identification of geriatric problems; health outcomes | ≥65 y  I: 356  C: 331 | ED LOS, h, mean | 4.9 | 3.9 | NC | S |
| Gagnon et al [29] (1999)  Canada | RCT;  10 mo | QoL; satisfaction with care; FS; HLOS; ED revisits | ≥70 y, with ADL needs, at risk for ED revisit  I: 212  C: 215 | ED revisits, within 10 mo post randomization (SD), mean | 1.2 (2.0) | 0.9 (1.2) | 0.18 (-0.01-0.37) | S |
| Lightbody et al [30] (2002)  UK | RCT;  6 mo | Falls; FS; ED revisits | ≥65 y, fall incident  I: 155  C: 155 | ED revisits, within 6 mo post index visit, n | 43 | 58 | 0.74 (0.54-1.03) | NS |
| McCusker et al [31] (2003)  Canada | RCT;  7-8 mo | Referrals and visits to community care services; ED revisits | ≥65 y  I: 166  C: 179 | ED revisit, within 1 mo post index visit, n | 58 | 48 | 1.30 (0.95-1.79) | NR |
| Mion et al [32] (2003)  USA | RCT;  8-9 mo | ED revisits; hospital or nursing home admissions; costs | ≥65 y  I: 326  C: 324 | ED revisit, within 1 mo post index visit, n | 66 | 49 | 1.34 (0.96-1.87) | NR |
|  |  |  |  | ED revisit, within 4 mo post index visit, n | 121 | 128 | 0.94 (0.77-1.14) | NR |
| Caplan et al [33] (2004)  Australia | RCT;  20 mo | All hospital admissions | ≥75 y  I: 370  C: 399 | ED revisits, within 1 mo post index visit, n | 42 | 51 | 0.89 (0.61-1.30) | NS |
|  |  |  |  | ED revisits, within 18 mo post index visit (SD), mean | 0.92 (1.3) | 1.1 (1.5) | -0.13 (-0.27-0.01) | NS |
| Guttman et al [37] (2004)  Canada | CBA;  12 mo | Unscheduled ED revisits | ≥75 y  I: 819  C: 905 | Unscheduled ED revisits, within 8 d post index visit, n | 69 | 104 | 0.73 (0.55-0.98) | NS* |
|  |  |  |  | Unscheduled ED revisits, within 14 d post index visit, % | 105 | 146 | 0.79 (0.63-1.00) | NS* |
| Lee et al [34] (2007)  Canada | RCT;  17 mo | Anxiety; fear of falling | ≥70 y, with fall incident  I: 43  C: 43 | ED revisits, within 2 mo post index visit, n | 8 | 8 | 1.00 (0.41-2.42) | NS |
| Courtney et al [35] (2009)  Australia | RCT;  40 mo | Emergency service use | ≥65 y, with medical diagnosis, at risk for ED revisit  I: 49  C: 58 | ED revisits, within 6 mo post index visit, n | 21 | 49 | 0.51 (0.36-0.71) | S |
| Mortimer et al [44] (2010)  Australia | NRCT;  2 mo | ED LOS | ≥65 y, with chronic disease; ≥70 y, without chronic disease  I: 101  C: 98 | ED LOS, h, mean | 12.7 | 10.1 | NC | S |
| Foo et al [38] (2012)  Singapore | CBA;  12 mo | Falls; FS; mortality | ≥65 y  I: 293  C: 169 | ED revisits, within 3 mo post index visit, n | 54 | 49 | 0.64 (0.45-0.89) | NR |
|  |  |  |  | ED revisits, within 6 mo post index visit, n | 94 | 69 | 0.79 (0.61-1.01) | NR |
|  |  |  |  | ED revisits, within 9 mo post index visit, n | 115 | 83 | 0.80 (0.65-0.99) | NR |
|  |  |  |  | ED revisits, within 12 mo post index visit, n | 126 | 90 | 0.81 (0.67-0.98) | NR |
| Mangram et al [39] (2012)  USA | CBA;  12 mo | Time efficiency | ≥60 y, with traumatic injury within previous 48 h  I: 393  C: 280 | ED LOS, within 12 mo pre/post intervention start, mean h | 4.2 | 6.1 | NC | S |
| Biese et al [36] (2014)  USA | RCT;  10 wk | Care plan compliance | ≥65 y, with outpatient follow-up needs  I: 45  C: 48 | ED revisits, within 35 d post index visit, n | 10 | 13 | 0.82 (0.40-1.68) | NS |
| Conroy et al [40] (2014)  UK | CBA;  24 mo | Admission avoidance from the ED | ≥65 y  I: 2,490  C: 2,184 | ED revisits, within 7 d, post index visit, n | 97 | 108 | 0.79 (0.60-1.03) | NR |
|  |  |  |  | ED revisits, within 1 mo, post index visit, n | 221 | 254 | 0.76 (0.64-0.91) | NR |
|  |  |  |  | ED revisits, within 3 mo d, post index visit, n | 456 | 482 | 0.83 (0.74-0.93) | NR |
| Keyes et al [41] (2014)  USA | CBA;  12 mo | ED revisits | ≥65 y  I: 3,748  C: 3,850 | ED revisits, within 1 mo, post index visit, n | 475 | 461 | 1.06 (0.94-1.19) | NR |
|  |  |  |  | ED revisits, within 6 mo, post index visit, n | 1,038 | 1,099 | 0.97 (0.90-1.04) | NR |
| Taylor et al [42] (2016)  UK | CBA;  2 mo | Time until geriatrician review in EAU | ≥75 y  I: 413  C: 398 | Time until geriatrician review in EAU, h, mean | 11 | 20 | NC | S |
|  |  |  |  | EAU revisits, within 7 d, post index visit, n | 31 | 28 | 1.08 (0.66-1.76) | NS |
|  |  |  |  | EAU revisits, within 1 mo, post index visit, n | 68 | 69 | 0.95 (0.70-1.29) | NS |
| *NRCT, non-randomized controlled trial; mo, months; y, years; I, intervention group; C, control group; RR, relative risk ratio; CI, confidence interval; ED, emergency department; LOS, length of stay; NC, not calculable; S, statistically significant; USA, United States of America; RCT, randomized controlled trial; QoL, quality of life; FS, functional status; HLOS, hospital length of stay; ADL, activities of daily living; mo, months; SD, standard deviation; UK, United Kingdom; n, number; NS, not statistically significant; NR, not reported; CBA, controlled before after; h, hours; wk, weeks; d, days; EAU, emergency assessment unit.*  **Based on unadjusted regression analyses.* | | | | | | | | |
