# Supplementary material for: Effectiveness of interventions to alleviate emergency department crowding by older adults: a systematic review
Source: BMC Emerg Med. 2019 Nov 20;19:69. doi: 10.1186/s12873-019-0288-4 (PMC6864956; doi:10.1186/s12873-019-0288-4)
Supplement: Supplementary file 3 — Additional file 3. Studies organized according to type of intervention and outcome. [file 12873_2019_288_MOESM3_ESM.doc]

| **Source (year)** | **Design** | **Intervention components** | | | | | | |  | **Outcomes** | | | **Risk of bias** |
| --- | --- | --- | --- | --- | --- | --- | --- | --- | --- | --- | --- | --- | --- |
|  |  | **ED-based GA** | **ED-based geriatric expertise** | **Geriatric emergency care area** | **Discharge instructions** | **Disposition planning** | **Follow-up care** | **MD case management** |  | **ED LOS** | **Time until geriatrician review** | **ED revisits** |  |
| **Hospital-based interventions** |  |  |  |  |  |  |  |  |  |  |  |  |  |
| Miller et al [43] (1996) | NRCT | √ | √ |  |  | √ | √ |  |  | ↑ | NR | NR | High |
| McCusker et al [31] (2003) | RCT | √ |  |  |  | √ | √ | √ |  | NR | NR | ↔ | High |
| Mion et al [32] (2003) | RCT | √ | √ |  |  | √ | √ | √ |  | NR | NR | ↔ | Moderate |
| Guttman et al [37] (2004) | CBA |  | √ |  | √ | √ | √ |  |  | NR | NR | ↔ | High |
| Courtney et al [35] (2009) | RCT |  |  |  | √ | √ | √ | √ |  | NR | NR | ↓ | Moderate |
| Mortimer et al [44] (2010) | NRCT |  | √ |  | √ | √ |  |  |  | ↑ | NR | NR | High |
| Foo et al [38] (2012) | CBA | √ | √ |  |  | √ |  | √ |  | NR | NR | ↔ | High |
| Mangram et al [39] (2012) | CBA |  |  | √ |  |  |  | √ |  | ↓ | NR | NR | High |
| Conroy et al [40] (2014) | CBA | √ | √ | √ |  |  |  | √ |  | NR | NR | ↔ | High |
| Keyes et al [41] (2014) | CBA | √ | √ | √ |  | √ |  | √ |  | NR | NR | ↔ | High |
| Taylor et al [42] (2016) | CBA | √ | √ | √ |  |  |  | √ |  | NR | ↓ | ↔ | High |
| **Community-based interventions** |  |  |  |  |  |  |  |  |  |  |  |  |  |
| Gagnon et al [29] (1999) | RCT |  |  |  |  | √ | √ | √ |  | NR | NR | ↑ | Low |
| Lightbody et al [30] (2002) | RCT |  |  |  |  |  | √ |  |  | NR | NR | ↔ | High |
| Caplan et al [33] (2004) | RCT | √ |  |  |  | √ | √ | √ |  | NR | NR | ↔ | High |
| Lee et al [34] (2012) | RCT |  |  |  |  |  |  |  |  | NR | NR | ↔ | Low |
| Biese et al [36] (2014) | RCT |  |  |  |  |  | √ |  |  | NR | NR | ↔ | High |
| *ED, Emergency department; GA, Geriatric assessment; MD, Multidisciplinary; LOS, Length of stay; NRCT; Non-randomized controlled trial; NR, Not reported; ↑, statistically significant effect in favor of the control group; ↔, no statistically significant effects between intervention and control group; ↓, statistically significant effect in favor of the intervention group; RCT, Randomized controlled trial; CBA, Controlled before after.* | | | | | | | | | | | | | |
